# Satellite-based time-series of sea-surface temperature since 1981 for climate applications

**DOI:** 10.1038/s41597-019-0236-x

**Published:** 2019-10-22

**Authors:** Christopher J. Merchant, Owen Embury, Claire E. Bulgin, Thomas Block, Gary K. Corlett, Emma Fiedler, Simon A. Good, Jonathan Mittaz, Nick A. Rayner, David Berry, Steinar Eastwood, Michael Taylor, Yoko Tsushima, Alison Waterfall, Ruth Wilson, Craig Donlon

**Affiliations:** 1 Department of Meteorology, University of Reading, Reading, UK; 2 National Centre for Earth Observation, University of Reading, Reading, UK; 3 Brockmann Consult GmbH, Hamburg, Germany; 4 National Centre for Earth Observation, University of Leicester, Leicester, UK; 5 Present Address: EUMETSAT, Darmstadt, Germany; 6 Met Office, Exeter, UK; 7 National Oceanography Centre Southampton, Southampton, UK; 8 MetNo, Oslo, Norway; 9 Centre for Environmental Data Analysis, Science and Technology Facilities Council Rutherford Appleton Laboratory, Harwell, UK; 10 Space Connexions Ltd., Harpenden, UK; 11 European Space Agency, Noordwijk, Netherlands

**Keywords:** Climate sciences, Physical oceanography

## Abstract

A climate data record of global sea surface temperature (SST) spanning 1981–2016 has been developed from 4 × 10^12^ satellite measurements of thermal infra-red radiance. The spatial area represented by pixel SST estimates is between 1 km^2^ and 45 km^2^. The mean density of good-quality observations is 13 km^−2^ yr^−1^. SST uncertainty is evaluated per datum, the median uncertainty for pixel SSTs being 0.18 K. Multi-annual observational stability relative to drifting buoy measurements is within 0.003 K yr^−1^ of zero with high confidence, despite maximal independence from *in situ* SSTs over the latter two decades of the record. Data are provided at native resolution, gridded at 0.05° latitude-longitude resolution (individual sensors), and aggregated and gap-filled on a daily 0.05° grid. Skin SSTs, depth-adjusted SSTs de-aliased with respect to the diurnal cycle, and SST anomalies are provided. Target applications of the dataset include: climate and ocean model evaluation; quantification of marine change and variability (including marine heatwaves); climate and ocean-atmosphere processes; and specific applications in ocean ecology, oceanography and geophysics.

## Abstract (summary)

**Table:** 

Measurement(s)	temperature of water
Technology Type(s)	satellite imaging
Factor Type(s)	time • geographic location
Sample Characteristic - Environment	sea
Sample Characteristic - Location	Earth (planet)

Machine-accessible metadata file describing the reported data: 10.6084/m9.figshare.9939638

## Background & Summary

Sea surface temperature (SST) is an “essential climate variable^1^”. Applications of SST data include the evaluation of climate and ocean models, observational quantification of climate change and variability, process understanding and parameterisation, ocean ecology, oceanography and geophysics. SST has been measured *in situ* for over 150 years^2^, initially from ships and in recent decades from drifting and moored autonomous platforms. SST products derived from Earth-orbiting satellites are complementary to the *in situ* network, providing finer and more complete spatio-temporal sampling. Satellite SSTs are indirect measurements (“retrievals”), inferred from at-satellite radiances by an inverse method.

**Fig. 1:**
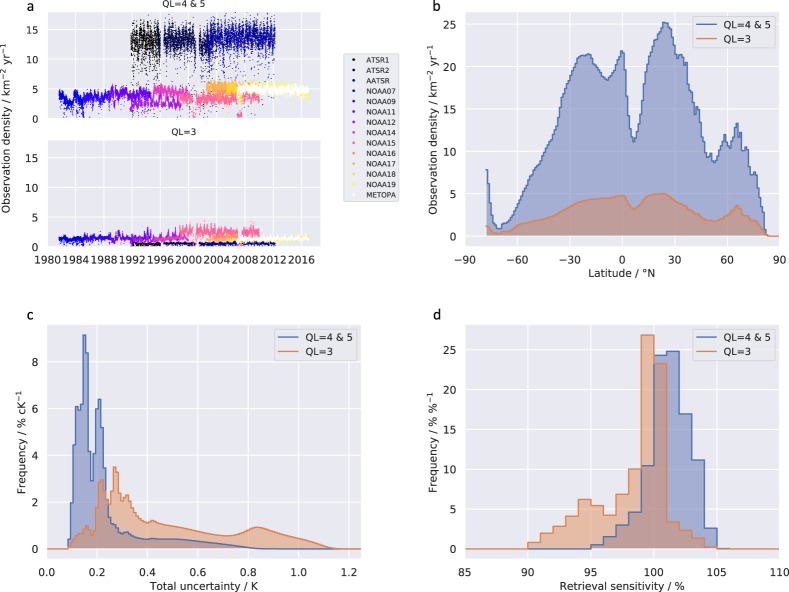
Characteristics of full resolution (L2P) CCI SSTs (derived from ATSR and AVHRR imagery). (**a**) Number of SST observations per unit area of ocean (“observation density”), per sensor over time. Each dot represents the observations of a single day. The upper panel shows data for the quality levels recommended for climate applications (QL 4 & 5), and the lower panel shows QL 3. (**b**) Zonal SST observation density per unit area, for all sensors combined and averaged over the time series. (**c**) Histogram of evaluated uncertainty in SST observations, for all sensors combined over the time series. (**d**) Histogram of evaluated sensitivity of SST observations, for all sensors combined over the time series.

This paper presents a climate data record (CDR) of global SST spanning 1981–2016 derived from 4 × 10^12^ satellite measurements of thermal infra-red (TIR) radiance. The TIR measurements were collected by two series of sensors on Earth-orbiting satellites: 11 Advanced Very High Resolution Radiometers (AVHRRs) and three Along-Track Scanning Radiometers (ATSRs). The spatial footprint of the TIR observations used for good-quality SST retrieval is between 1 km × 1 km (the resolution of the ATSR imagery at nadir view) and 18 km × 2.5 km (the maximum footprint of AVHRR global area coverage (GAC) pixels used). Valid SSTs are obtained from TIR measurements with cloud-free views of ice-free ocean. Quality levels (QLs) are provided that reflect an assessment of the validity of each datum and its uncertainty. QLs are on a scale 0 to 5 inclusive, and values of 4 and 5 are recommended for climate applications. The number of such SST estimates obtained is 13 km^−2^ yr^−1^ on average. The SST observation density varies in time and space (Fig. 1).

Data are provided in four forms: at their native resolution (orbit view); in files of grid-cell-mean SST at 0.05° latitude-longitude resolution (data for individual sensors, as uncollated individual orbits, or collated as daily combinations); and as a blended multi-sensor and gap-filled product on a daily 0.05° grid. In the international nomenclature of satellite processing levels^3^, these versions of the CDR consist respectively of datasets of the following types: level-2 pre-processed (L2P), level-3 uncollated (L3U), level-3 collated (L3C) and level-4 analysis (L4).

**Fig. 2:**
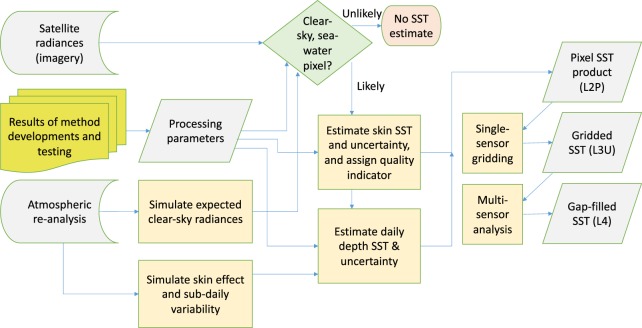
Overview of logic of the production of climate data records (CDRs) for sea surface temperature (SST). Calibrated, geo-located satellite data comprising radiance (or brightness temperature) imagery form the fundamental source data. Many processing parameters have been defined by physics-based algorithm development, addressing the harmonisation of satellite radiances between sensors (i.e., consistency of calibration), probabilistic (Bayesian) pixel classification to determine for which pixels valid SSTs can be obtained, retrieval of the skin SST (to which thermal radiances are directly related), adjustment of skin SST to estimate daily mean SST at a nominal depth comparable to *in situ* measurements, and estimation of SST retrieval and sampling uncertainty. The retrieval and skin-to-depth adjustment steps are informed by estimates of the past atmospheric conditions at the observation locations from numerical weather prediction re-analysis outputs. The low-level SST CDR is at the full pixel resolution of the input imagery (L2P), is gappy because of clouds, and comprises SST and its per-pixel uncertainty. L2P SSTs are averaged on a grid of 0.05° in latitude and longitude to obtain L3U products, sensor by sensor, with propagated cell-mean uncertainty. L3U SSTs are combined across many sensors to generate an interpolated, gap-filled L4 analysis, with uncertainty estimates.

Figure 2 presents the overview of the logic of the production of the CDRs, including the relationships of the different product levels.

SST values at all levels in the CDR have associated with them a per-datum evaluation of standard uncertainty^4^. In level 2 and 3 products, a decomposition of the total uncertainty into components with differing correlation structures is also provided. The evaluated total uncertainty is less than 0.8 K for almost all SSTs, and the median evaluated uncertainty for L2P SSTs (i.e., for individual ATSR and AVHRR retrievals) is 0.18 K. The multi-annual global observational stability for the time series, relative to drifting buoy SSTs is, with 95% confidence, in the range −0.0026 to 0.0004 K yr^−1^. Taken together, these statistics suggest that the dataset gives a detailed representation of SST variability on a range of space and time scales of relevance to climate applications.

SSTs derived from IR radiances are sensitive to the variation in temperature of the skin layer of the ocean^5^. The skin SST is the temperature most appropriate for determining instantaneous air-sea fluxes, since skin SST determines the surface radiative cooling of the ocean and the temperature and humidity of the air in contact with the air-sea interface. For many purposes, SST estimated at a depth below the skin effect is more appropriate. The difference between skin and depth SST is typically of order tenths of kelvin, but can be larger^5^. *In situ* SST measurements^2^ and the upper layers of ocean models typically reflect SST at depths between ~10 cm and ~10 m. In order to use satellite SSTs with the centennial SST record^6^, estimates comparable to depths sampled by ships’ buckets^7^ and drifting buoys are needed. Here, adjustments are provided to convert the instantaneous skin SST to a depth of 20 cm, nominally corresponding to drifter and historic bucket temperature measurements.

**Fig. 3:**
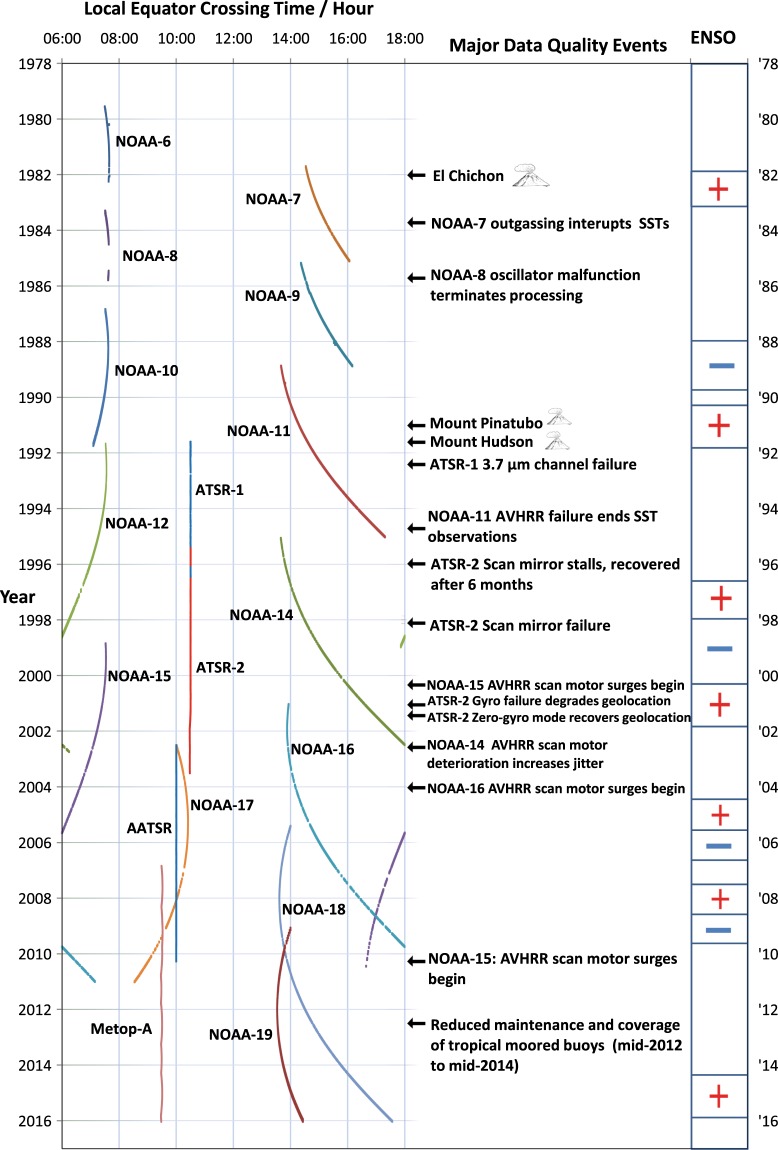
Timeline of contributing missions, geophysical and on-orbit events affecting data quality, and El Nino Southern Oscillation (ENSO) phase.

Satellite local overpass times differ between missions and sometimes drift during missions (Fig. 3). The diurnal cycle in sea surface temperature has been empirically characterised from sub-daily drifting buoy variability^8^ and by remote sensing^9^, and is typically in the peak-to-peak range of 0.1 K to 0.5 K. Under low-wind, strong-sun conditions, it can be ~5 K^10^. Different overpass times differentially sample SST, generating non-climatic signals if not adjusted for. For this reason, the depth adjustment mentioned above also addresses the diurnal cycle, the depth SST being further adjusted in time to be more representative of the daily mean. Such an adjustment for skin-to-depth and local-time-of-day effects has only, to our knowledge, been done for this CDR and its precursors. The L4 analysis represents a multi-satellite estimate of daily mean SST at 20 cm depth.

By using ATSR series sensors as the calibration reference for the CDR, a distinctive objective for our CDR is to be as independent of *in situ* observations as possible. A high degree of independence is achieved for the period 1995 to the end of the record, exploiting ATSR-2 and AATSR. Prior to that period, it has been necessary in this v2.1 CDR to use *in situ* SSTs as a “calibration” reference on large scales, so that to a significant degree, independence from *in situ* measurements is lost when considering the period 1981 to 1995.

## Methods

The dataset is the cumulative outcome of more than a decade of methodological development of: Bayesian methods of cloud screening of imagery^11,12^; harmonisation of sensor calibrations^13^, inversion of TIR radiances to SST independently of *in situ* measurements (i.e., based on physical modelling not empirical tuning^14^), physical modelling of time-of-day adjustments of retrieved SSTs to minimise the aliasing of daily SST cycles into long-term trends^15^, and context-specific estimation of total SST uncertainty and uncertainty components^16,17^. For the purpose of this project, the most complete possible collection of AVHRR GAC data has been assembled.

### Input data

The nature of the satellite datasets used in this work is summarised in Table 1. The ATSR-series data (ATSR, ATSR2 and Advanced ATSR) consist of the entire v3/v2.1 level-1b archive (http://data.ceda.ac.uk/neodc/aatsr_multimission/). The level-1b designation indicates that these data consist of calibrated, geo-located brightness temperatures and radiances. The full archives of the AVHRR-series GAC data (AVHRR 7, 9, 11, 12, 14, 15, 16, 17, 18 and 19) were sourced from the “CLASS” archive of NOAA together with additional orbits from the University of Miami (AVHRR 7, 9, 11, 15, 16). GAC data include instrument counts that need to be converted to calibrated radiances. For reflectance wavelengths, a previously published approach to calibration (“PATMOS-X”) is adopted^18^, which has a stated uncertainty of 2% across sensors. For the thermal channels, AVHRRs brightness temperatures are re-calibrated on-orbit (see below under ‘Harmonisation’) and improvements to flagging of solar contamination events are implemented.

**Table 1:** Summary characteristics of satellite level-1 data used.

Sensor	Date range used (year/month/day)	Typical local time of observation	0.6 μm	0.8 μm	1.6 μm	3.7 μm	11 μm	12 μm
AVHHR-7	1981/08/24 to 1985/02/18	15.00 h*	Y	Y	N	Y	Y	Y
AVHRR-9	1985/01/04 to 1988/11/07	15.00 h*	Y	Y	N	Y	Y	Y
AVHRR-11	1988/10/12 to 1994/09/13	15.00 h*	Y	Y	N	Y	Y	Y
AVHRR-12	1991/09/16 to 1998/12/14	07.00 h*	Y	Y	N	Y	Y	Y
ATSR-1	1991/11/01 to 1996/01/09	10.30 h	N	N	2Y	2Yf	2Y	2Y
AVHRR-14	1995/01/19 to 1999/12/31	15.30 h*	Y	Y	N	Y	Y	Y
ATSR-2	1995/08/01 to 2003/06/22	10.30 h	N§	N§	2Y	2Y	2Y	2Y
AVHRR-15	1998/09/24 to 2009/12/31	06.00 h*	Y	Y	N	Y	Y	Y
AVHRR-17	2002/07/10 to 2009/12/31	10.00 h*	Y	Y	Yd	Yn	Y	Y
AATSR	2002/07/24 to 2012/04/08	10.00 h	2Y	2Y	2Y	2Y	2Y	2Y
AVHRR-16	2003/06/01 to 2006/12/31	16.00 h*	Y	Y	Yd	Yn	Y	Y
AVHRR-18	2005/06/05 to 2009/12/31	13.30 h*	Y	Y	Yd	Yn	Y	Y
AVHRR-19	2009/02/22 to 2016/12/31	13.30 h*	Y	Y	Yd	Yn	Y	Y
AVHRR Metop-A	2006/11/21 to 2016/12/31	09.30 h	Y	Y	Yd	Yn	Y	Y

For some sensors the date range used is less than the period of data delivery, because data used has been truncated for quality reasons. The local time of observation varies significantly for sensors whose indicative time is marked with an asterisk (*). Y or N refers to the presence/use of a waveband. 2Y indicates dual-view observation at a waveband (near-nadir and slanted along track). § indicates that data availability over the oceans was restricted and the channel was not used. Some channels are provided only for day or night scenes (with variations in regards to which is available for twilight scenes), which is indicated by Yd and Yn respectively. The ATSR-1 channel marked 2Yf failed early in the mission, but is used where present.

Figure 3 indicates events with significant geophysical signatures in SST and events that affect data quality for individual sensors and, in the case of the major volcanic eruptions, all IR sensors then observing.

Numerical weather prediction (NWP) fields are used as auxiliary information for cloud detection and retrieval. We use the European Centre for Medium-range Weather Forecasting Re-Analysis Interim (ERA-Interim) dataset^19^, which is consistent in that it is generated with a single version of the atmospheric general circulation model and assimilation scheme, although the availability of data sources for the assimilation evolves through the period. Sea-ice concentration from the Ocean and Sea Ice Satellite Application Facility (OSI-SAF) is used within processing for screening of ice-covered seas, and is processed and provided to users for convenience in the L4 analysis product.

### Auxiliary data

Land-sea boundaries are determined from the results of ESA’s land-cover (LC) CCI project^20^. The LC CCI classifications were additionally processed to create distance-to-land and water-body-identifier datasets^21^, a derived distance-to-land raster at 1/120^th^° which we use to assess whether the field of view of a given satellite radiance is wholly filled with water, given its centre location and its view angle. Grid cells at 0.05° latitude-longitude resolution are designated as “ocean” if they are partially ocean. The Caspian Sea is included as ocean.

Static pre-calculated look-up tables (LUTs) are referenced during cloud detection. The cloud detection auxiliary files quantify the conditional probability density function for observed multi-channel wavelength combinations, as a function of parameters such as satellite view angle, given the condition that the observed area is cloud-affected. Separate LUTs are defined for AVHRR and ATSR series.

LUTs are also pre-calculated for the SST retrieval applied to the ATSR-series imagery. These LUTs consist of retrieval coefficients (see ‘Retrieval methods’ below). Spectral response functions for all ATSR-series and AVHRR-series sensors are used in the radiative transfer simulations that underpin both cloud detection and retrieval. The land-sea mask, cloud LUTs, retrieval coefficients and spectral response functions used are available at 10.5281/zenodo.2586714.

Three major volcanic eruptions (El Chichon^22^; Pinatubo and Hudson^23^) caused two periods of elevated stratospheric sulfate aerosol (1982–84, 1991–93) with impacts on infra-red brightness temperatures that are significant for SST retrieval^24^ (e.g., >0.03 K). For cloud detection and SST retrieval from AVHRR, it is useful to have a prior measure of stratospheric aerosol loading and its uncertainty as it evolves in time. We derived an auxiliary dataset for this from High-Resolution Infrared Radiation Sounders by adapting a published method^25^.

### Radiance harmonization

Harmonisation is addressed at the level of infra-red radiance (or, equivalently, brightness temperature). Harmonisation is the reconciliation of the in-flight calibration of sensors, accounting for their measured differences in spectral response^26^. This reconciliation is achieved by re-calibration of sensors against a reference channel. The reference for channels centred near 3.7 µm and 11 µm is the Advanced ATSR (AATSR). The reference for channels centred on 12 µm is ATSR-2. These choices reflect our level of confidence in the spectral response information and on-board calibration characterisation across the constellation of sensors. In general, we have most confidence in the AATSR among the available sensors. However, the 12 µm channel of AATSR was subject to an anomalous bias of up to 0.3 K, which we have reduced by application of a shift of its nominal spectral response function^27^, therefore for that channel we have more confidence in ATSR-2. Coincidences (within a space-time window) are used between AATSR and ATSR-2 from the period during which both were observing. The expected differences in brightness temperatures (found by radiative transfer modelling accounting for spectral response differences) are compared with those observed, and an empirical parameterisation of the unexplained differences is found, effectively bringing the calibration of the two sensors into alignment. A similar process applies to the 11 µm and 12 µm channels of ATSR-1, which are harmonised to the equivalent channels of ATSR-2. No reference for the 3.7 µm channel of ATSR-1 is available because the channel failed early in the mission.

The approach is somewhat different for AVHRRs, for which the coefficients of the counts to radiance conversion are re-evaluated, which is harmonisation by re-calibration. The reference sensors for the AVHRR are the ATSR-2 and AATSR. For re-calibrating across sensors, we gathered a dataset of sensor-sensor match-up data consisting of equal-zenith-angle views of a common location within a 5 minute time window. Discrepancies in ATSR/AVHRR BTs (having taken account of expected differences given the available spectral response functions by radiative transfer simulation) are minimised in a least-squares sense by re-estimating the AVHRR coefficients for counts-to-radiance conversion. Outside the period of the reference sensors, overlaps between AVHRRs are similarly used to obtain a chain of calibrations.

### Radiative transfer modeling

Cloud detection and retrieval are based on the physics of radiative transfer. Two radiative transfer models are used in the project.

SST retrieval coefficients for the ATSRs are derived from line-by-line layer-by-layer simulations of top-of-atmospheric spectral radiance performed at a channel-dependent spectral resolution always finer than 0.6 × 10^−3^ cm^−1^. Except for some differences explicitly described below, the methods are based on published approaches^14^. The radiative transfer model used is LBLRTM^28^ v12.2, with the AER^29,30^ v3.2 spectroscopic databases. Simulated spectral radiances are convolved with ATSR-series spectral response functions to obtain channel radiances. Tropospheric aerosol absorbing and scattering effects are addressed by perturbing channel radiances. Simulations are performed on 2,100 training locations distributed across seasons and across the global oceans with adequate and balanced sampling of profiles geographically, seasonally and with respect to surface temperature and total column water vapour (TCWV). The atmospheric profiles input to LBLRTM comprise meteorological (dynamic) variables and secular (composition) variables. The meteorological variables are air temperature and humidity, and skin surface temperature. Trace gases are included which have absorption properties relevant to simulation of thermal window channels. The trace gas concentrations evolve in time in order to ensure that their secular trends do not cause trend artefacts in SST retrievals.

SST retrieval for the AVHRRs is based on fast radiative transfer modelling (also used for cloud detection). “Fast” here means that channel-integrated radiative transfer is highly parameterised. We use the model RTTOV^31^ version 11.3 for calculating and integrating clear-sky absorption and (for infrared) emission of channel radiance. Surface reflectance and emission are calculated using respective modules specifically defined for the ocean surface interfaced to RTTOV. The surface emissivity module is a function of wavelength, view angle, windspeed, temperature and salinity, derived from modelling sea-surface wave-facet slope distribution and optical properties^32,33^.

### Cloud detection

Clouds absorb radiance emitted from the sea surface and emit radiance at the cloud top temperature. SST retrieval under the assumption of cloud-free conditions is therefore erroneous if pixels are in fact fully or partially cloud filled. Cloud detection is applied to the satellite imagery to minimise cloud biases in SSTs. Cloud-affected radiances differ from clear-sky radiances because of contrasting spectral emissivity, spectral reflectance, spectral brightness temperature and/or spatial coherence. The same applies to sea-ice affected radiances. For identifying clear-sky pixels, we calculate the probability of clear-sky given the radiances and the prior atmospheric and surface state using Bayes’ theorem as follows^11,12^:

$$P(c| {\bf{y}},{\bf{x}})=\frac{P({\bf{y}}| {\bf{x}},c)P({\bf{x}}| c)P(c)}{P({\bf{y}}| {\bf{x}})P({\bf{x}})}$$

where: *c* is the condition of being clear-sky over ice-free ocean; **y** is the observation vector, here containing the brightness temperatures (BTs) of thermal channels, the reflectances (for day-lit scenes) of reflectance channels and a local standard deviation of BT over 3-by-3 pixels of a thermal channel; and **x** is the state vector, listing variables describing the prior understanding, from NWP, of the surface temperature, surface wind speed, atmospheric temperature profile and atmospheric humidity profile. This expression simplifies assuming *P*(**x**|*c*) = *P*(**x**), since the background state has length scales of ~100 km and does not resolve cloud structures at the finer scales ~1 to ~10 km relevant to the cloudiness of individual pixels. In practice, the term *P*(**y**|**x**) is evaluated as $P({\bf{y}}| {\bf{x}})=P(\bar{c})P({\bf{y}}| {\bf{x}},\bar{c})+P(c)P({\bf{y}}| {\bf{x}},c)$, where the over-bar indicates “not clear-sky over water” and $P\left(\bar{c}\right)=1-P\left(c\right)$. Evaluating the posterior probability of clear-sky therefore amounts to quantifying: *P*(*c*), the prior probability of a pixel being clear-sky; *P*(**y**|**x**, *c*), the probability density function (pdf) of the observation vector, given the NWP and a condition of clear skies; and $P\left({\bf{y}}| {\bf{x}},\bar{c}\right)$, the pdf of the observation vector given cloud conditions. For *P*(*c*), the NWP local cloud fraction is used, although constrained to the range 0.05 and 0.5 so as not to determine the outcome too strongly from that prior. *P*(**y**|**x**, *c*) is calculated on-the-fly by radiative transfer simulation, accounting for the uncertainty in **x**, noise in observations and uncertainty in forward modelling. $P\left({\bf{y}}| {\bf{x}},\bar{c}\right)$ is evaluated from look-up tables, obtained iteratively by accumulating the reflectance, brightness temperature and spatial coherence properties of cloud-flagged areas over several years of orbits in a prior pass of cloud detection; for this purpose, AATSR and the AVHRR on Metop-A are used to create pdf LUTs used for all the sensors in their respective series.

SSTs are evaluated for those pixels for which the posterior probability of clear sky, *P*(*c*|**y**, **x**), exceeds 90% (case of ATSR series) or 99% (case of AVHRR series). The probability does not have a frequentist interpretation (i.e., when *P*(*c*|**y**, **x**) = 90% visual inspection suggests that the image pixels are cloudy less than 10% of the time).

### Retrieval of skin SST

Sea surface temperature retrieval relies on the sensitivity of top-of-atmosphere radiances to the Planck emission from the sea surface. Because of the sea surface’s non-ideal spectral emissivity and because of absorption, emission and scattering processes in the atmosphere, BTs differ from the underlying SST. For the “window channels” generally used for SST retrieval—namely, 11 µm, 12 µm and (for night-time scenes) 3.7 µm—magnitudes of SST-BT difference for different thermal channels and view angles bear relationships that allow multi-channel observations (and multi-angle observations where available) to be inverted to estimate the SST. A variety of inverse algorithms have been published and reviewed^34,35^.

Single-pixel SSTs from the ATSR-series are derived using retrieval coefficients that weight the observed dual-view brightness temperatures using the equation

$$x={a}_{0}+{{\bf{a}}}^{\text{T}}{\bf{y}}$$

where *x* is the retrieved SST, *a*_0_ is an offset coefficient, **a** is a vector listing weights for each channel brightness temperature (BT) and **y** is the observation vector containing the corresponding channel brightness temperatures. For ATSR SSTs, the observation vector includes BTs at both nadir (0° to ~22°) and forward (~53°) view angles. The coefficients are defined for different strata of total column water vapour (TCVW) and are smoothly interpolated to the prior TCWV obtained from the NWP fields interpolated at the time and place of the observation. Likewise, the coefficient LUTs have dimensions in nadir and forward view angle, and in time (to account for the secular evolution of trace gases). These single-pixel SSTs are used to form the higher-level products, beginning with the L3U product, which contains the simple averages of clear-sky single-pixel SSTs in 0.05° grid-cells.

The products containing full resolution SST imagery (L2P) from the ATSR-series are populated with atmospherically smoothed SST estimates. Atmospherically smoothed SSTs are preferable for ATSR SSTs to reduce the SST noise at full spatial resolution. SSTs from dual-view retrievals can yield very low uncertainty from systematic effects but are prone to being noisy (relatively large independent random errors in individual pixel SSTs), particularly when using two channels (at 11 and 12 µm). For the imagery products, it is useful to reduce this noise somewhat by exploiting the fact that the space scales of the clear-sky atmosphere tend to be much longer than the SST pixel separations. The retrieval method is a variant of the single-pixel method shown above:

$$\mathop{x}\limits^{ \sim }={a}_{0}+{{\bf{a}}}^{\text{T}}\langle {\bf{y}}\rangle +{{\bf{b}}}^{\text{T}}({\bf{y}}-\langle {\bf{y}}\rangle )$$

where $\widetilde{x}$ is the atmospherically smoothed^36^ SST for the central pixel of a 5 × 5-pixel box. **y** is the vector of BTs spatially averaged across the clear-sky pixels of that box. The retrieval coefficients, *a*_0_ and **a**, are as for single-pixel SST retrieval. The vector **b** has unit magnitude, its contents are non-zero only for nadir channels, and the non-zero terms are inversely proportional to the square of each channel’s radiometric noise. The smoothed retrieval thus comprises a box-average SST plus a term that adjusts for within-box variability of SST to make a lower-noise estimate of the SST of the pixel at the centre of the box.

Single-pixel SSTs, rather than atmospherically smoothed, are used as input to the L3U product since averaging to 0.05° in any case averages down the independent random noise, and the propagation of uncertainty from single-pixel SSTs is simpler.

SSTs from the AVHRR-series are derived using an atmospherically smoothed reduced-state-space pseudo-maximum-likelihood inverse^37^. Unlike the ATSR-series, the AVHRRs are single-view sensors, which reduces the information content available for determining SST compared to the dual-view ATSRs. Particularly for day-lit scenes, where only the 11 and 12 µm channels are used, single-view coefficient-based retrievals are associated with geographical biases arising from this information content deficit^38^. We use an inverse within the family of “optimal estimation” (OE) algorithms to bring additional prior information to the retrieval explicitly. The retrieved state vector is a reduction of the full state profile to three summary terms: ${\bf{z}}={[x,\bar{x},\bar{w}]}^{\text{T}}$ where $\bar{x}$ and $\bar{w}$ are SST and TCWV averaged over the surrounding clear pixels of the 3 × 3 GAC box centred on the pixel for which the retrieved SST is *x*. The smooth-atmosphere-but-variable-SST assumption is imposed by fixing the TCWV for the centre pixel and the surrounding clear pixels to be identical (hence only one TCWV term in **z**). $\bar{x}$ emerges from the calculation but is not used. The OE approach is based on the difference between the observations and simulated BTs derived from RTTOV applied to the full prior-state profile from NWP. Designating the simulated BTs as **F**(**x**), we have

$${\bf{z}}={{\bf{z}}}_{a}+{{\bf{S}}}_{a}{{\bf{K}}}^{\text{T}}{({{\bf{K}}{\bf{S}}}_{a}{{\bf{K}}}^{\text{T}}+{{\bf{S}}}_{\varepsilon })}^{-1}({\bf{y}}-{\bf{F}}({{\bf{x}}}_{a}))={{\bf{z}}}_{a}+{\bf{G}}({\bf{y}}-{\bf{F}}({{\bf{x}}}_{a}))$$

where **x**_*a*_ is both a prior estimate of the state and point of linearization for forward modelling; **z**_*a*_ is the reduced equivalent to **x**_*a*_; **S** variables are error covariance matrices, ${{\bf{S}}}_{\varepsilon }$ being that of the measurement-relative-to-forward-model errors, and **S**_*a*_ being that of the reduced prior state errors; **K** comprises the derivatives of the observations in **y** with respect to the reduced state variables, which are outputs of RTTOV. A crucial choice is the magnitude of uncertainty attributed to the prior SST (which in the NWP system was obtained from a number of operational sources^19^). Since the prior NWP fields have some dependence on *in situ* SST, we choose to minimise the influence of the prior SST on the retrieved state by adopting an inflated uncertainty in the prior SST that is sufficiently small to provide useful regularisation of the inverse and sufficiently large that the influence of the prior on the retrieved SST is controlled to be <5% for the SSTs given a quality indication of 4 or 5 (see below). Sensitivity to true SST is estimated as the leading term in the “averaging kernel” matrix **GK**, and quantifies the fractional response in the retrieved estimate to true SST variability. The sensitivity is associated with each SST, and the median sensitivity for QL 4 & 5 data is 101%, close to the ideal value of 100%. Thus, a high level of independence from *in situ* SST observations is preserved, despite use of prior information for the above inverse.

### Uncertainty estimate for skin SST

Estimates of standard uncertainty^4^ (which may be considered as the standard deviation of the estimated error distribution) are provided for every SST^39^ at all product levels. Errors in satellite-derived SSTs do not all fall neatly into those arising from random and systematic effects, since errors introduced in the retrieval are locally correlated between pixels^40^. For each skin SST estimate in the L2P products, a total uncertainty estimate is provided, which is the standard uncertainty from all sources of error combined. The total uncertainty is derived by combining three components of uncertainty, whose estimates are also provided. The components are designated by their error correlation structure (uncorrelated, synoptically correlated and large-scale correlated).

Errors that are independent (uncorrelated) between observations arise from the instrumental noise in the satellite observations of brightness temperature. The uncorrelated component of uncertainty is estimated therefore by propagating models of instrumental noise through the retrieval process. Typically, the process of retrieval amplifies noise by a factor that varies between ~2 and ~8, depending on the channel combination, viewing geometry and atmospheric state.

The component of uncertainty labelled as synoptically correlated refers to errors that are largely in common (nearly perfectly correlated) between SST observations that are adjacent and simultaneous, and become randomised (uncorrelated) as spatio-temporal distance between observations increases. The decorrelation length scales are not yet quantified in detail, but the physical origin of the correlated errors is understood to arise from the imperfectly accounted-for influence of the atmospheric state on the estimated SST^41^. In the case of coefficient-based retrievals, the uncertainty is estimated from the residuals when determining the coefficients. In the case of optimal estimation, the error covariance matrix of the retrieval is a standard quantity that is calculated, and extracting the component corresponding to the propagation of **S**_*a*_ through the retrieval provides an estimate of the SST uncertainty. Since the effect causing the errors reflects aspects of the atmospheric state, the decorrelation scales are related to the length scales of the atmosphere, and are considered likely to be of order 1 day and 100 km.

The systematic component in the SST uncertainty covers all effects that may be described as biases, whether in the sensors’ calibrations, radiative transfer models or physical assumptions made in retrieval (for example, in relation to the loading of atmospheric aerosol). This component of uncertainty is difficult to estimate, although an upper bound of order 0.1 K can be established by comparison with other SST data, and this value is used.

Some sources of error are not estimated by the above procedures. These include the SST impact of cloud-affected pixels that nonetheless pass the cloud-detection procedures, unaccounted-for aerosol effects on BTs from volcanic eruptions and mineral dust, and issues such as undetected solar contamination of measurements that may affect AVHRRs. It is for these reasons that an indicator of quality is also given. Evidence of an observation context in which true uncertainty may be significantly larger than evaluated is one factor in downgrading the quality level attached to an SST.

### Quality indication

A confidence level on a scale 0 to 5 is provided for each SST as a quality indicator, following an international convention^3^. Five (5) indicates the highest confidence. Quality levels 4 and 5 should be used for climate applications where absolute accuracy of SST is important. Some users may find lower quality level data useful, e.g., where SST front locations are detectable in the SST fields, which requires only relative, not absolute, accuracy.

The quality indicator is influenced by the confidence we have that the SST uncertainty estimate for a given SST is valid^39^. SSTs with relatively high uncertainty can still therefore be flagged as good quality, provided there is nothing to indicate that the assumptions made in estimating the uncertainty are compromised. The most significant quality factors are undetected cloud and coarse-mode aerosol (primarily desert dust): the uncertainty estimates for SST are valid under clear-sky, low-aerosol conditions, and therefore the quality level 5 is attributed only for high clear-sky probability and for conditions of low aerosol (assessed by a desert dust index^42,43^) or where steps to adjust for aerosol are taken (the case for the volcanic stratospheric aerosol events). The desert dust index is only available to check for the ATSR series sensors, since it relies on having dual-view observations. Users need to be aware that mineral-dust-affected AVHRR SSTs are present intermittently in the products, particularly for the north east tropical Atlantic Ocean, the Red Sea and the Gulf of Arabia, and such data may be given quality level 5 flags without the biasing effect of the aerosol being accounted for in the attached uncertainty; see further information in Usage Notes below. This is an aspect of the dataset that requires improvement in future work.

In the case of optimally estimated SSTs, the goodness-of-fit of posteriori simulated and observed brightness temperatures is calculated, using a chi-square statistic. Large values of chi-square indicate that the inter-relationships of the brightness temperatures are not as expected for a clear-sky observation given the background information. The quality levels of pixels with large chi-square are therefore downgraded.

In order to maximise the use of this dataset for assessment of *in-situ* based SST measurements and for model testing, it is important for the SSTs to have high sensitivity to true SST variations (which means minimal dependence on the prior SST information). For this reason, the quality levels of SSTs with low sensitivity are downgraded.

**Fig. 4:**
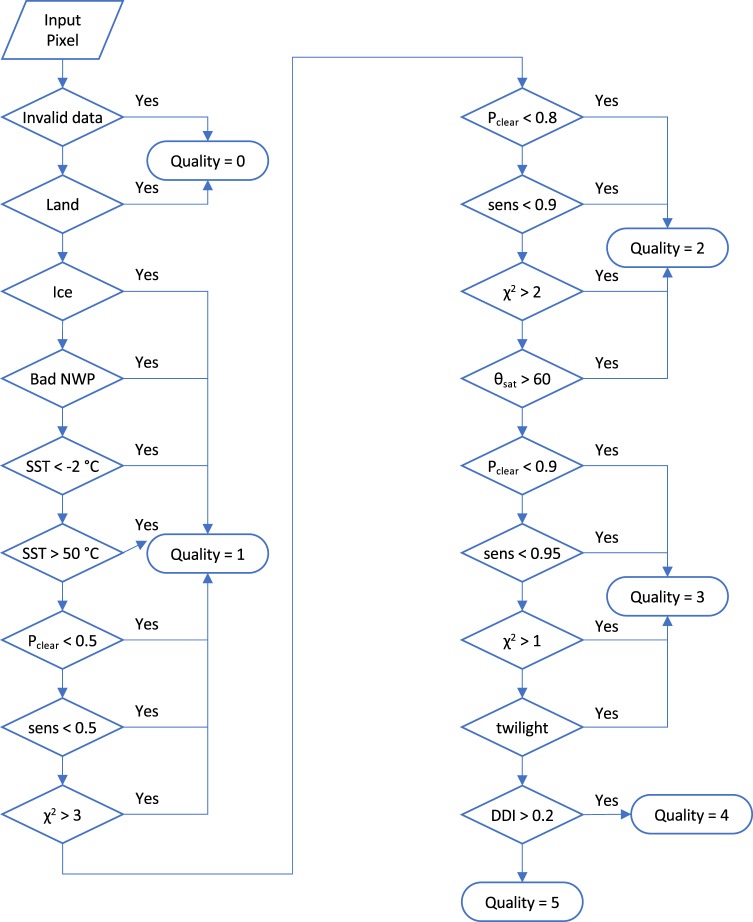
Logic and thresholds for assigning pixel quality level (QL) flags to L2P SSTs. “Invalid data” means input data are flagged as invalid. “Land” means the centre of the pixel is not over ocean. “Bad NWP” means tests on the integrity of the prior fields are failed. P_clear_ is the posterior probability of the pixel being clear-sky over ice-free ocean. “sens” is the evaluation of sensitivity of the retrieval to true changes in SST. *χ*^2^ is the channel-normalised result of a test on the goodness of fit achieved in retrieval (applies to optimally estimated SST only). *θ*_sat_ is the satellite zenith angle of the retrieval. “twilight” means the solar zenith angle at the target pixel is within 5° of 90°. “DDI” refers to an index for presence of desert (mineral) dust aerosol (available for ATSRs only). The P_clear_ threshold of 0.90 for designation as QL > 3 is for the ATSRs and AVHRRs at night-time, and for AVHRRs observations during daytime is instead 0.99.

The thresholds and logic for quality level assignment are shown in Fig. 4.

### Adjustments by depth and time

The primary retrieved quantity is the skin SST estimate made at the satellite overpass time. The skin SST is pertinent to air-sea fluxes, but nonetheless, many users seek an estimate of SST at depths of order tens of centimetres, whether because this makes the observations compatible with many historic *in situ* SSTs from drifting buoys and bucket measurements, or because this depth is more comparable to the upper layer of an ocean model. For this reason, the products include an adjustment which, when added to the skin SST, gives an estimate of the SST at a depth of 20 cm. By adding this adjustment, the resulting SST is nominally comparable to what would be measured by a drifting buoy at the satellite observation time.

There is a diurnal cycle in SST that has been empirically characterised using satellite observations^9^ and drifting buoys^8^. The satellite observations are obtained at local times of day that change through the record (Fig. 3 and Table 1). Aliasing of this diurnal cycle with varying times of observation will produce spurious inter-annual trends if not adjusted for. For this reason, an adjustment is also calculated for time-of-day effects. The SST at 10.30 or 22.30 local mean solar time is a good approximation to the daily mean^8^. Moreover, from 1991 onwards, there has always been a mid-morning satellite observing at close to this local time needing minimal adjustment. For these reasons the temporal adjustment is an estimate of the change in SST between the observation time and the nearest of 10.30 or 22.30 local mean solar time. The time and depth adjustments are estimated using a one-dimensional turbulence closure model driven by re-analysis surface fluxes and wind stress. The uncertainty from this adjustment is also calculated and included in the total uncertainty provided for the daily mean depth SST estimate.

### Gridded SST products

**Fig. 5:**
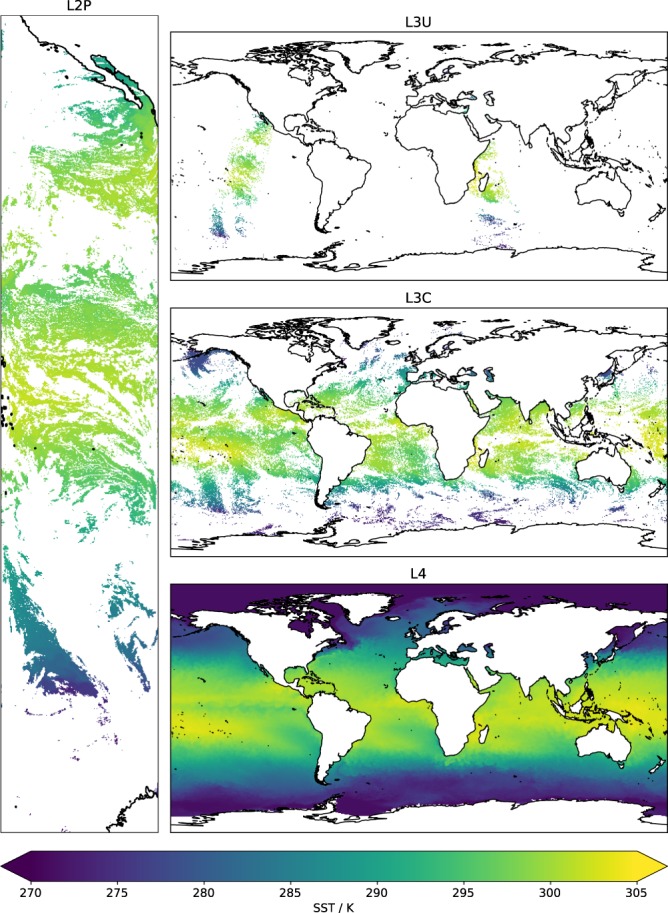
SST content in data in different product levels. L2P data are on the original viewing geometry, with gaps in SST from cloud cover. These are gridded per orbit to L3U. A day’s worth of L3U SSTs from a given sensor is collated to form an L3C product. Data from multiple sensors are merged and interpolated to give the daily gap-free SST field of the L4 analysis.

Gridded versions of data (L3U and L3C, see Fig. 5) are provided on a spatial grid of 0.05° in latitude and longitude. Gridded L3U products are made from L2P (full resolution orbit data) by averaging only the SSTs of the highest available quality level within the cell. Simple averaging is used. The quality level of the gridded value is the quality level of the data used to form the average.

When averaging *n* L2P SSTs to make daily 0.05° gridded L3 products, the uncertainty from random errors decreases from “1/√*n*” averaging, whereas the uncertainty from the other two components does not. (When gridding L2P data to larger and/or longer scales, averaging down of the correlated errors would occur, but this is negligible for one pass on a scale of the grid cell.) The SST of a 0.05° cell is often calculated from pixels that do not fill the cell, because of cloud cover, but users typically treat the gridded SST as a value representative of the cell as a whole, and therefore the sub-sampling is another source of uncertainty. The uncertainty is parameterised effectively in terms of the fraction of the cell observed and the variability in SST in the observed part of the cell^17^. There is no correlation of this effect between cells, so this contributes to the uncorrelated component of uncertainty in the L3U SST products.

Each day’s L3U SSTs from each individual sensor are gathered as L3C SST (gridded daily products). The content of an L3C grid cell is the average of the highest quality L3U SST obtained during the day.

### Analysed SST

The gridded SST products are used as the inputs to a gap-filled estimate of the daily-mean SST field on the same 0.05° grid (Fig. 5). This L4 product is intended to represent the SST at 20 cm depth, and the time-and-depth adjusted SSTs are the inputs used. The method of estimating the spatially complete SST field is variational assimilation, using a scheme called NEMOVAR^44^. The principle of the variational assimilation scheme is to minimise a cost function

$$J(\delta {\bf{x}})\propto \delta {{\bf{x}}}^{\text{T}}{{\bf{B}}}^{-1}\delta {\bf{x}}+\delta {{\bf{y}}}^{\text{T}}{{\bf{R}}}^{-1}\delta {\bf{y}}$$

with respect to *δ***x**, which is the change between the present-day’s solution and a forecast of the present day based on the previous-day’s solution. **B** represents the error covariance of the forecast. *δ***y** is the difference between the new SSTs observed during the present day and the expected SST observations given the solution *δ***x**. Even if the new solution were perfect, *δ***y** would be non-zero because of uncertainty (SST measurement errors and representativity effects), and **R** represents the error covariance associated with that uncertainty. For computational simplicity, the errors in the new SSTs are assumed to be uncorrelated within the analysis system. The solution found by the minimum therefore balances the information carried forward from the previous day with the information added by new observations, in the light of their relative uncertainty, accounting for correlations in the forecast errors.

A key aspect of the variational assimilation scheme is the parameterisation of **B**, which affects the location and degree of smoothing of SST features that inevitably occurs when in-filling the gappy observations of SST of a given day. **B** influences the feature resolution of the analysis. In the scheme used, **B** is parameterised such that the degree of smoothing reacts to the local variability of SST (quantified by the SST gradients present in the previous-day’s solution)^45^. Note that the feature resolution is thus coarser than the grid cell size. Uncertainty information is also provided in the L4 products, which is estimated using an analysis quality method^46^.

## Data Records

The dataset title, digital object identifier, full name, description, and data volume are given for our products^47–53^ at different processing levels in the Tables 2 to 5. All data are released under the licence Creative Commons Attribution 4.0 International (CC-BY 4.0, https://creativecommons.org/licenses/by/4.0/).

**Table 2:** Data record information for orbit-geometry (level 2) SST CCI products.

Full dataset title	ESA SST CCI ATSR L2P v2.1^47^	ESA SST CCI AVHRR L2P v2.1^48^
Full name	European Space Agency Sea Surface Temperature Climate Change Initiative: Along-Track Scanning Radiometer level-2 pre-processed product version 2.1	European Space Agency Sea Surface Temperature Climate Change Initiative: Advanced Very High Resolution Radiometer level-2 pre-processed product version 2.1
Basic description (quotable when citing data)	global sea surface temperatures from Along-track Scanning Radiometers, presented on the native geometry of observation at original time of observation, and spanning 1991 to 2012	global sea surface temperatures from Advanced Very High Resolution Radiometers, presented on the native geometry of observation at original time of observation, and spanning 1981 to 2016
Total data volume	2.6 T	5.4 T
Recommended acronym/short name, when referring to product	SST CCI ATSR	SST CCI AVHRR
Recommended acronym/short name, when referring to SST in product	CCI ATSR SST	CCI AVHRR SST

**Table 3:** Data record information for gridded, single-sensor (level 3U) SST CCI products.

Full dataset title	ESA SST CCI ATSR L3U v2.1^49^	ESA SST CCI AVHRR L3U v2.1^50^
Full name	European Space Agency Sea Surface Temperature Climate Change Initiative: Along-Track Scanning Radiometer level-3 uncollated product version 2.1	European Space Agency Sea Surface Temperature Climate Change Initiative: Advanced Very High Resolution Radiometer level-3 uncollated product version 2.1
Basic description (quotable when citing data)	global sea surface temperatures from Along-track Scanning Radiometers, presented on a 0.05° latitude-longitude grid at original time of observation, and spanning 1991 to 2012	global sea surface temperatures from Advanced Very High Resolution Radiometers, presented on a 0.05° latitude-longitude grid at original time of observation, and spanning 1981 to 2016
Total data volume	270 G	3.1 T
Recommended acronym/short name, when referring to product	Gridded SST CCI ATSR	Gridded SST CCI AVHRR
Recommended acronym/short name, when referring to SST in product	CCI gridded ATSR SST	CCI gridded AVHRR SST

**Table 4:** Data record information for gridded, single-sensor collated (level 3C) SST CCI products.

Full dataset title	ESA SST CCI ATSR L3C v2.1^51^	ESA SST CCI AVHRR L3C v2.1^52^
Full name	European Space Agency Sea Surface Temperature Climate Change Initiative: Along-Track Scanning Radiometer level-3 collated product version 2.1	European Space Agency Sea Surface Temperature Climate Change Initiative: Advanced Very High Resolution Radiometer level-3 collated product version 2.1
Basic description (quotable when citing data)	global sea surface temperatures from Along-track Scanning Radiometers, daily collations on a 0.05° latitude-longitude grid, and spanning 1991 to 2012	global sea surface temperatures from Advanced Very High Resolution Radiometers, daily collations on a 0.05° latitude-longitude grid, and spanning 1981 to 2016
Total data volume	242 G	2.8 T
Recommended acronym/short name, when referring to product	Gridded daily SST CCI ATSR	Gridded daily SST CCI AVHRR
Recommended acronym/short name, when referring to SST in product	CCI gridded daily ATSR SST	CCI gridded daily AVHRR SST

**Table 5:** Data record information for the gridded, multi-sensor, interpolated (level 4) SST CCI product.

Full dataset title	ESA SST CCI Analysis v2.1^53^
Full name	European Space Agency Sea Surface Temperature Climate Change Initiative: Analysis product version 2.1
Basic description (quotable when citing data)	global daily-mean sea surface temperatures, presented on a 0.05° latitude-longitude grid, with gaps between available daily observations filled by statistical means, spanning 1981 to 2016
Total data volume	414 G
Recommended acronym/short name, when referring to product	SST CCI analysis
Recommended acronym/short name, when referring to SST in product	CCI analysis SST

## Technical Validation

### Verification processes

For all levels of SST CCI data in each public release, technical verification and quality tasks are undertaken, consisting of three steps: automated inspection of all files; visual inspection of a random subset; and manual verification of metadata against the product specification document (PSD)^54^.

The automated quality assessment of L2P and L3U consists of checks that are configurable for each product level and are applied to every file in the dataset. The checks consist of basic checks (file name follows the convention, file can be read, etc) and specific checks on the data (all variables exist, data are within prescribed boundaries, etc). Specific checks include the verification of inter-variable consistency, i.e. if a pixel contains a fill-value in the SST variable, the associated uncertainty variables must also contain a fill-value at the same location. The consistency of per-pixel flags versus per-pixel quality indicators is verified: e.g., quality level 0 (“no_data”) should be present for pixels flagged to be over land. The automated verification results are stored per-file and merged into a summary, ensuring traceability of the results. A set of standardised graphics for each sensor is generated that facilitates a global view of the data quality.

For the visual inspection, two products of each sensor and processing level are randomly selected, one from early and one from late in the sensor’s useful life. Using the toolbox SNAP each variable in this file set is visually inspected for artefacts, unusual structures or inconsistent geometries. The histogram of each variable is checked for plausibility. Manual verification of the product metadata against the PSD is done using two randomly selected products from the dataset for each processing level. Global metadata and the attributes of each variable are checked against the prescribed values defined in the PSD. Visual inspections of L4 files were also undertaken.

### Verification results

The automated inspection was applied to all 465,302 L2P SST products and the same number of L3U SST products. The total data volume analysed comprises 8 TB of L2P and 3.4 TB of L3U data. The results of the automated inspection of the dataset show a high degree of technical compliance. Two non-compliances have been detected, as follows. Around 0.005% of the SST files contain only fill value data; spot checking shows that these files originate from periods of outgassing or are satellite commissioning-phase acquisitions. 0.001% to 0.5% of records (depending on the sensor) show flag or mask inconsistencies; these inconsistencies only appear at quality level 1 (“bad_data”), and so do not affect the recommended uses of the dataset; these inconsistencies will be resolved in a future version.

The visual inspection of the test-dataset did not reveal any unusual structures. All histograms of the variables showed the expected distributions. Minor discrepancies between data and product user guide have been detected that will do not affect the usability of the dataset.

### Inter-comparison

A Climate Assessment Report^55^ presents an assessment of trends and variability in the SST CCI products (at all levels: L2P, L3 and L4) and comparison to other SST products. In order to assess the multi-annual and decadal behaviour of the new products, comparisons are made to existing long-term (usually coarser resolution) SST data sets used in high profile monitoring reports. Differences between the SST CCI products and the comparison datasets are highlighted. The SST CCI products are also assessed against previous releases by the ESA CCI SST project to determine what progress has been achieved. This process is not validation, but does provide important context for potential users to allow them to determine whether or not the products are credible CDRs and might prove useful.

Time series of SST anomalies referenced to a long-term climatology are calculated and compared for 61 regions of the world’s oceans, together with relevant indices, such as for the El Nino Southern Oscillation. Linear trends in these regional series are presented. Maps of decadal average anomalies demonstrate any large-scale differences between the new products and the comparison data sets. Maps of correlations at different lags demonstrate the level of persistence seen in the products. Should these diagnostics then highlight anything worth exploring further, bespoke investigations can be made.

The Group for High-Resolution SST (GHRSST) Multi-Product Ensemble (GMPE) system was designed to allow intercomparison of near real time analyses^56^. The GMPE system regrids all the input data on to a common 0.25° grid and generates the median and standard deviation of the analyses available on each day. Daily files are generated containing the median and standard deviation, as well as the differences between each individual analysis and the GMPE median. In addition, a map of gradients in the SST analyses (calculated on their original grids and regridded to the standard GMPE grid) is provided. This analysis is also included in the Climate Assessment Report and provides a mechanism for comparison of the SST CCI analysis product to other higher-resolution analyses (largely) for the satellite era, alongside the comparison to longer-term data sets outlined above.

### Validation against *in situ* measurements

**Fig. 6:**
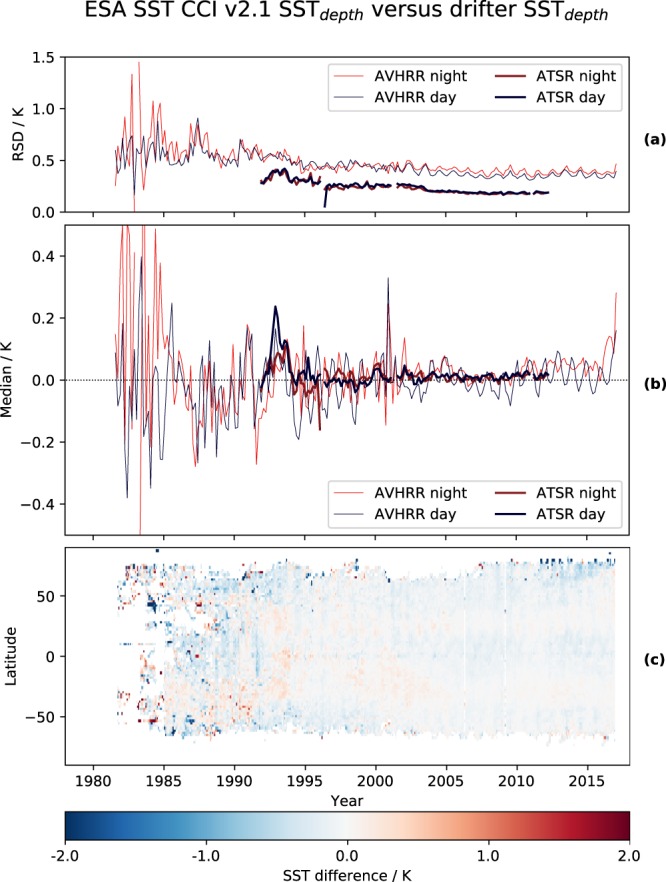
Overview of validation results. Robust standard deviation (**a**) and median discrepancy (**b**) for comparison of CCI ATSR and AVHRR SSTs with drifting buoys. Depth-depth validation results are shown, in which satellite skin SSTs have been adjusted to 10:30 am/pm local time and *in situ* records have been temporally interpolated to the same time. (**c**) Time/latitude variation of analysis minus drifting-buoy SST differences, averaged zonally and monthly.

All products have been validated against *in situ* measurements of SST according to the Product Validation Plan^57^. In interpreting validation results, the degree of independence between the measurements being compared is important. To further secure the objectivity of validation results, the personnel performing the validation analysis were independent of the teams undertaking the remote sensing research and product generation. Three categories of validation were carried out. ‘Skin-raw’ comparisons direct compared skin SSTs from the satellites with matched *in situ* data, not attempting to adjust for the known geophysical processes that give rise to differences. In ‘skin-skin’ validation, the *in situ* data are adjusted to the skin-depth and time of the matched satellite measurement. In ‘depth-depth’ validation, the satellite retrieval adjusted to 20 cm depth at 10:30 h or 22.30 h local time is compared to *in situ*. Depth and time adjustments were calculated using a combined model of the skin-effect and diurnal thermocline^15^. The validation analysis was done for all levels (L2P, L3U and L3C) for both the SST CCI ATSR and SST CCI AVHRR records. The SST CCI analysis (in which no *in situ* measurements are assimilated) was also validated ‘depth-depth’, in accordance to the definition of the analysis. Full detailed validation results for individual sensors contributing to the SST CCI ATSR and SST CCI AVHRR will be published elsewhere. An overview of results from validating the SST CCI ATSR and SST CCI AVHRR records against drifting buoys (‘depth-depth’) is shown in Fig. 6. The statistics shown are robust standard deviations (RSD, equal to the scaled median absolute deviation, the scaling being chosen to match the standard deviation for a normal distribution) and median discrepancies between CCI and *in situ* SSTs. Figure 6c is the variability of the median discrepancy against drifting buoys over time and latitude for the SST CCI analysis. The excellent stability of the ATSR series observations, especially for the ATSR-2 and AATSR periods, is emphasised in panel b. CCI AVHRR SSTs are also generally better during the period of overlap with ATSRs from the 1990s onwards. Note that some of larger discrepancies during the 1980s reflect the relative sparsity and quality of the *in situ* network at that time, as well as artefacts in the CCI SSTs discussed further in the usage notes that follow. Regional variability in the SST CCI AVHRR results in latitudes affected by dust aerosols manifest as a cool bias in the SST CCI analysis results in the zone from 0° to 20° N.

## Usage Notes

### Fitness-for-purpose assessment (climate science applications)

Prior to public release, the data have been used in climate modelling experiments at the Met Office Hadley Centre and by a number of trail-blazer users given early access in exchange for feedback. A brief conceptual summary of these trial uses follows.

CCI analysis SSTs were used as the lower-boundary forcing in atmosphere-only simulations and compared with compatible simulations forced with HadISST.2.2.0.0 daily ¼° SST^58^. The impacts on simulation of cloud regimes, tropical cyclones, the Asian summer monsoon, the Madden-Julian Oscillation, the El Nino Southern Oscillation (ENSO) and mid-latitude storm tracks were all objectively assessed. The differences were generally smaller than differences arising from changes in model resolution. Beneficial and detrimental differences were observed. Warmer SST around the Maritime Continent and as well as cooler SST around the equatorial Atlantic and upwelling regions of at the eastern boundaries of ocean basins were associated with reduced bias in cloud regimes, although inference that the CCI analysis SSTs are more realistic cannot be directly made. The CCI analysis SSTs tend to be warmer than the comparator in the Southern Hemisphere, and there is southward shift in the distribution of the tropical organized convective regime and a corresponding increase in simulated tropical cyclone activity in that hemisphere, although the impact was modest compared to existing model biases. In the monsoon regions, colder Arabian Sea SSTs in the L4 analysis reduced the moisture flux over the western Ghats mountains, and there were detrimental increases in rainfall (from increased convergence) in the South China Sea and western Pacific Ocean, related to warmer SSTs in these regions.

Trail-blazer users looked at various applications. An application of the data to provide an SST climatology for the Australian seas found the data to be highly consistent with mooring measurements in the region and able to provide a convincing climatology. A study on the use of CCI analysis SSTs at the locations of coral reefs near Florida and Belize found that the inferred coral-reef heat stress could differ from previous estimates^59^. An assessment of CCI analysis SSTs for oceanographic application over the Eastern Atlantic concluded that open ocean SST values had lower uncertainties than in coastal zones, where comparisons with coastal buoys gave discrepancies of between 0.3 K and 0.8 K (root mean square differences), concluding that higher feature resolution would benefit near-coastal applications. A study of ENSO variability in a coupled climate model showed reduced cold-tongue simulation biases from a model upgrade, relative to SST CCI observations.

Overall, notwithstanding the limitations of the SST CCI products identified by inter-comparison and validation, users found the datasets easy to use and useful within the context of their applications. Users of a precursor version of SST CCI data have demonstrated its use for evaluating biases between different instrumentally homogenous observational datasets^60^ and for propagating observational uncertainty to scales required in model evaluation^61^.

Known artefacts in the CDR v2.1 include the following. Unscreened and unadjusted-for desert dust events cause intermittent negative biases of magnitude 1 K in CCI AVHRR SSTs in the north east tropical Atlantic, Red Sea and Gulf of Arabia; the sensitivity of ATSR-series sensors to these events is much less. Since only AVHRRs are available during the first decade of the CDR, whereas ATSRs were available from 1991 to 2012, the CCI analysis SST anomalies show an exaggerated positive trend in these regions through the time series, thought to be about 0.01 to 0.02 K yr^−1^. During the 1980s, we were able to use only one AVHRR sensor at a time other than brief overlaps. Some periods of degraded calibration of these sensors cause temporary observational instabilities in the CDR. The following periods show biases not accounted for within the stated uncertainties that introduce artefacts in the global-mean CCI analysis SST appearing to be in the range of 0.1 to 0.5 K: May 1982, October to December 1982, early August 1983 and late September 1983.

### Reading the products (quick start)

All data are stored in NetCDF-4 format files. Data arrays in NetCDF files are known as ‘variables’ and each variable has metadata stored with it. To get correct values in correct units, the add_offset and scale_factor attributes need to be applied when reading the variables; many tools will do this automatically for NetCDF files, so no action may be necessary. The names of key variables in the product files are given in Table 6 below. The notes below the table include important points about interpreting the quality and location.

**Table 6:** Key information about variables in SST CCI products.

Description of the content of key variables in the NetCDF files	Names of variables in files containing single (L2P) or gridded (L3U) orbits of data	Names of variables in files containing merged multi-sensor (L4) data
Latitudes of the data points	lat	lat
Longitudes of the data points	lon	lon
Sea surface temperature at the skin* using best available retrieval	sea_surface_temperature	N/A
Total uncertainty of the sea surface temperature at the skin*^,#^	sea_surface_temperature_total_uncertainty	N/A
Sea surface temperature at 20 cm depth and 10.30 am or pm local time	sea_surface_temperature_depth	N/A
Infilled daily-mean estimate of sea surface temperature at 20 cm depth	N/A	analysed_sst**
Total uncertainty of the sea surface temperature at 20 cm depth^#^	sea_surface_temperature_depth_total_uncertainty	analysed_sst_uncertainty
Sea surface temperature anomaly at 20 cm depth	sea_surface_temperature_depth_anomaly	
Quality level	l2p_flags	
Location type	N/A	mask****
Fractional coverage of sea ice	N/A	sea_ice_fraction

*Skin SST is the temperature of the radiating surface layer of the water, which is of order 10 µm depth.

**‘Analysis’ is the term used for the combination and interpolation of the SSTs from the orbit files; an alternative set of products is available where this variable is replaced with analysed_sst_anomaly.

***Good quality SSTs are those where the value in the SST data array is not −32768 and the value in the quality_level variable is 4 or 5.

****Ice-free ocean SST values have mask = 1.

^#^Also available in the files is uncertainty broken down into different components.

### ATSR, AVHRR or analysis product?

A visual impression of what to expect in terms of SST data in different products is given by Fig. 6. To work on SST features such as fronts and eddies at the highest possible resolution, L2P products should be used, the disadvantage being the need to work with gappy data on non-repeating latitude-longitude co-ordinates. To work on data on a regular grid, but maximally preserving features, L3C products should be used, bearing in mind these are also gappy data. If spatially complete fields are required, the L4 analysis should be used. Users of L4 should bearing in mind that that this product is derived from the SSTs that are adjusted to 20 cm and to a local time representative of the daily average SST, and that the process of interpolation inevitably means feature resolution is degraded relative to the lower-level data.

### Which type of SST?

The sea_surface_temperature variable contains the primary observed quantity, which is the estimated temperature of the radiometric skin layer of the ocean at the time observed. This SST is the more directly relevant to instantaneous air-sea interactions. The sea_surface_temperature_depth variable contains an SST adjusted from the observed value to be more comparable with sub-surface *in situ* measurements (such as underpin centennial-scale SST reconstructions) and more representative of the daily mean SST (adjusting for time-of-day effects). This SST is more directly relevant for analyses of long-term SST differences and changes.

### How should I use the quality and uncertainty information?

Quality 4 and 5 SSTs should be used where the absolute accuracy of the SSTs is important, particularly for climate applications. Quality 3 SSTs may be usable by users to whom maximising the SST coverage is the primary concern, in applications (such as pattern-based analyses) where absolute accuracy is less critical.

The evaluations of total uncertainty provided are relevant to all users for propagating the SST uncertainty through their application and assessing the robustness of their findings. Examples of usage are available in a product user guide (available along with other documentation at http://www.esa-sst-cci.org).

Where applications involve use of the aggregated data on spatio-temporal scales coarser than the CCI SSTs, the errors contributing to the total uncertainty cannot be assumed to be fully independent between SST values. To assist users seeking to understand uncertainty in quantities derived from SSTs at other spatio-temporal scales, three components contributing to the total uncertainty are evaluated (in the L2P and L3C products). The large-scale correlated component can be treated as arising from “systematic” errors. The uncorrelated component describes uncertainty arising from independent (often called “random”) errors. The third component represents uncertainty arising from errors that are correlated locally – i.e., the errors are the same or similar for SSTs obtained near each other in space and time but become independent for large separations. The scales of correlation are not yet fully understood. However, a rule of thumb is that this component can be treated as “systematic” for scales less than ~100 km and ~1 day and “random” for scales much greater than these scales. Further work is required to develop more rigorous means of evaluating uncertainty across spatio-temporal scales.

### How should I refer to the products in publications?

Experience shows that it is sometimes difficult even for the data producer to infer which dataset has been used in publications based on previous data releases. We recommend to users the following practice, in reference to tables Tables 2 to 5. In the first reference to the dataset in a publication, the dataset title and/or full dataset name should be given, including version number (v2.1), which unambiguously identifies the dataset. A brief description of the dataset contents and characteristics can be based on the basic descriptive text suggested in Tables 2 to 4. When referring to the product thereafter, usage such as (from Table 2) “using the SST CCI ATSR products” is recommended. When referring to the SSTs in a product, usage such as (from Table 4) “frontal features in CCI analysis SSTs were stronger” is recommended. (“CCI” could be omitted if no similar products of different origin are used.) We encourage re-statement of the data version number in legends of figures, captions, presentation slides or other elements of publications that may circulate independently. Publications should reference this paper and the data citation. Following these suggestions will maximise the traceability and reproducibility of the work.

### Will the climate data record be extended in time?

The SST CCI v2.1 climate data record described here covers the period to the end of 2016. Products were generated using fixed processing configurations and auxiliary information. Under funding of the Copernicus Climate Change Service (C3S), the extension of SST data in time is ongoing, covering the start of 2017 onwards. The extension is an interim climate data record (ICDR): this means that the scientific basis and practical form are consistent, so that users can validly use the ICDR seamlessly with the longer dataset. Users should be aware that, in an ICDR, some parameters of the processing have to change over time or may not be fully optimised. For example: the NWP data stream used as auxiliary information must change in the ICDR during 2019, because of the scheduled halt to the production of ERA-Interim; instrument degradation may prompt us to halt using a satellite data stream or introduce a new data stream, but the timing of this may not be optimised as effectively as in a retrospective CDR reprocessing. Users will find that the ICDR consists of v2.0 files rather than v2.1. The main differences are that SST anomaly values are not precalculated and available for users in v2.0 files as they are in v2.1, and the uncertainty variables follow a different naming convention. Nonetheless, the SSTs are recommended for seamless use across the v2.0 ICDR and v2.1 CDR, since the scientific basis is fully consistent. The v2.0 ICDR will be available with the ongoing post-2016 extension products via the climate data store of the C3S, at https://cds.climate.copernicus.eu.

The European Space Agency recently funded a continuation of the SST CCI project, which will enable future release of SST CCI v3 products covering the period up to the end of 2020.

## Data Availability

For the toolbox SNAP see http://step.esa.int/main/toolboxes/snap/. Example code to read data products and generate Fig. 5 is available^62^.
